# Preparation of Lignosulfonates from Biorefinery Lignins by Sulfomethylation and Their Application as a Water Reducer for Concrete

**DOI:** 10.3390/polym10080841

**Published:** 2018-07-30

**Authors:** Caoxing Huang, Junmei Ma, Weiyu Zhang, Guohong Huang, Qiang Yong

**Affiliations:** 1 Co-Innovation Center for Efficient Processing and Utilization of Forest Products, College of Chemical Engineering, Nanjing Forestry University, Nanjing 210037, China; hcx@njfu.edu.cn (C.H.); junmeima123@163.com (J.M.); wyzhang180912@163.com (W.Z.); ghhuang@nhri.cn (G.H.); 2 State Key Laboratory of Pulp and Paper Engineering, South China University of Technology, Guangzhou 510640, China; 3 Department of Material and Structural Engineering, Nanjing Hydraulic Research Institute, Nanjing 210037, China

**Keywords:** biorefinery lignin, sulfomethylation, lignosulfonate, water reducer, concrete

## Abstract

Lignosulfonate (LG), a water-soluble polymer from sulfite pulping process of lignocellulosic biomass, has been commercially applied as admixture for concrete. In this work, lignosulfonates were produced from alkaline lignin (AL) and enzymatic hydrolysis residue (EHR) by sulfomethylation and these lignosulfonates as water reducers for concrete were then evaluated. Results showed that 94.9% and 68.9% of lignins in AL and EHR could be sulfonated under optimum sulfomethylation conditions, respectively. The sulfonic groups in lignosulfonates from AL (AL-LG) and EHR (EHR-LG) were 1.6 mmol/g and 1.0 mmol/g, respectively. Surface tension and zeta potential analysis indicated that both AL-LG and EHR-LG can be potentially used to as dispersant for improving the fluidity of the cement paste, similarly to commercial lignosulfonate (CM-LG). Adding 0.2 wt % of AL-LG, EHR-LG, and CM-LG in the concrete, the compressive strength (28 days) of concretes increased from 38.4 Mpa to 41.6, 42.6, and 40.9 Mpa, respectively. These findings suggest that the lignosulfonate from biorefinery lignin by sulfomethylation can meet the industrial standards as water reducers for cement admixtures.

## 1. Introduction

Lignin is the world’s most abundant naturally phenolic polymer, serving a variety of integral functions for non-woody and woody biomass [1]. Processed lignin can be obtained from biomass pulping industries, where it is usually regarded as a value-adding byproduct [2,3]. Unlike the lignin derived from pulping and papermaking processes, biorefinery lignins are produced from biorefinery processes which generate energy, fuels, and materials from renewable lignocellulosic biomass [4]. Existing pulp and paper operations have specific usages for their lignin by-products (e.g., fuel source and chemical recovery in kraft, value-adding lignosulfonates in sulfite), but biorefinery processes have yet to implement any sort of strategy for dealing with the lignin by-products that they produce [4,5]. Therefore, unique valorization of the lignin generated from lignocellulosic biorefinery process is essential to establish economic viability of such processes.

In the bamboo industry in China, a glut of bamboo residues solids is discarded due to their inferior mechanical properties [6]. While, only 30% of these residues are valorized as energy or used in production of activated carbon [7]. Therefore, utilization of the remaining bamboo residues is an urgent issue for the industry. Bamboo residues has been regarded as the potential material for the bio-based products by biorefinery process. During the biorefinery process, alkali-based pretreatments (kraft cooking and sodium hydroxide extraction) are the effective technologies for bamboo residues to prepare the digestible solid by enzyme for fermentable sugars production [8,9,10]. The solubilized lignin in alkali-based pretreatment liquor can be obtained by acidified precipitation, termed as alkaline lignin (AL). After enzymatic hydrolysis, lignin is left behind lignin-rich solid substrate, termed as enzymatic hydrolysis residue (EHR) [11]. For the biorefinery lignins of AL and EHR, both of them are required properly valorized application.

The potential application for lignin have been investigated in many fields. For example, valorizing lignin as thermoplastics, hydrogels, thermosets, antioxidants, coatings, lubricants, and surfactants have been explored for many years [5,12,13,14]. However, most of these explorations are still in various stages of research due to various process complications and high production costs. The most commercially-accepted lignin for several applications is lignosulfonate (LG), which has been commercialized for many decades [15,16]. Lignosulfonates are a class of modified lignin molecules which are produced from sulfite pulping of lignocellulosic biomass. Production of lignosulfonate via sulfite pulping is limited, and in some countries sulfite pulping operations are non-existent. Hence, an increasing amount of studies focusing on sulfonation or sulfomethylation of lignin have been performed for generating lignosulfonates [17,18]. Although sulfonation is a viable chemical pathway for preparing lignosulfonates from various industrial lignins, this chemical modification requires acidic conditions. As a negative, acidic conditions can also render undesirable inter-lignin condensation reactions, lowering the application viability of the sulfonated lignin product. In contrast, sulfomethylation of lignin can be carried out at various pHs. Due to its robustness, sulfomethylation has garnered increasing attention as a means for valorizing lignin sources.

Lignosulfonates have been used across a variety of applications, such as dye dispersants, chelating agents, and water reducers for cement [19,20]. Water reducers are a class of ingredients industrially used to increase the fluidity of cement admixtures, resulting in lowered water usage for concrete setting. This reduction in water translates to improved finished concrete strength [21]. The mechanism for how water reducers function is through imparting electrostatic repulsion and steric hindrance between cement particles, lowering admixture viscosity [19,22]. Because lignosulfonate is an anionic surface-active polymer capable of exerting electrostatic repulsion and steric hindrance, it is often applied as a water reducer for concrete admixtures. For the lignosulfonates from the lignin by sulfomethylation, it also can be used as the concrete admixtures. For example, Ouyang et al. [23] used a sulfomethylation technology on alkali lignin and revealed that the resulting lignosulfonate functioned as a concrete dispersant. In another work, Konduri and Fatehi [17] and He and Fatehi [18] used sulfomethylation to prepare sulfomethylated kraft lignin as a dispersant for cement admixture and found the resulting lignosulfonate improved the fluidity of a cement paste more effectively than lignosulfonate. Within the previously referred works, lignosulfonate generated from kraft lignin by sulfomethylation technology served as an effective dispersion effect to the cement paste. To our knowledge, few systematic studies have been done to evaluate whether lignosulfonates derived from the biorefinery lignin of bamboo residues can function as water reducers for concrete. For that reason, we have attempted to fill the knowledge gap by generating lignosulfonates from bamboo residues and evaluating their viability as water reducers.

The present study investigated systematically the performance of sulfomethylation on the production of lignosulfonate from biorefinery lignin (AL and EHR). The sulfonic group, molecular weight, surface tension, and zeta potential of lignosulfonate from AL and EHR were characterized. In addition, the potential application of these lignosulfonate as water reducer for concrete was evaluated by measuring the fluidity of the cement paste, water reducing rate, and compressive strength of concrete.

## 2. Materials and Methods

### 2.1. Materials

AL and EHR from alkali-pretreated bamboo residues were obtained through the processing described in our previous work [14,24]. The main chemical components of AL and EHR were quantified according to the laboratory analytical procedure from the National Renewable Energy Laboratory [25] and results are as follows: 94.2% and 64.9% of total lignin, 3.4% and 27.9% of glucan, and 0.4% and 7.4% of xylan (respectively). Commercial lignosulfonate (CM-LG) was purchased from Shanghai Tingnuo Materials Co., ltd. (shanghai, China).

### 2.2. Sulfomethylation of Alkaline Lignin (AL) and Enzymatic Hydrolysis Residue (EHR)

Sulfomethylation of AL and EHR were conducted according to the work of sulfomethylation of softwood and hardwood kraft lignin [17,18]. During sulfomethylation, 1 mol of formaldehyde mixed with 1 mol of sodium sulfite generate 1 mol of sodium hydroxymethylsulfonate—the reactive reagent for sulfomethylation. In this work, sulfomethylation of AL and EHR were conducted under different sodium hydroxymethylsulfonate/lignin ratios (0.1 mol/mol–1.4 mol/mol), temperatures (80–130 °C), and time intervals (1–5 h) within a stainless steel bomb reactor. Both of these sulfomethylation conditions were referred to the works of Konduri and Fatehi [17], He and Fatehi [18] who also used sulfomethylation to prepare lignosulfonates from hardwood and softwood kraft lignin. After the reaction, the hot reactor was cooled down immediately in an ice bath, and the contained sulfomethylation liquor (lignosulfonate solution) was neutralized to pH 5 by adding 1 M sulfuric acid to precipitate the un-reacted lignin. The remaining solid and lignosulfonate in sulfomethylation liquor was then separated by centrifuge. Finally, the remaining solids were dried at 105 °C overnight to calculate the amount of undissolved lignin. The lignosulfonate yield was analyzed based on the mass balance, as follows

(1) $$\mathrm{ignosulfonate}\;\mathrm{yield}=(1-\frac{W-W_{1}}{W})\times 100\%$$

where *W* is the amount of lignin in original solid (KL and EHR), *W*_1_ is the amount of lignin in the remaining solid after sulfomethylation. The amounts of lignin in *W* and *W*_1_ were analyzed according to the procedure developed by National Renewable Energy Laboratory (NREL) [25].

At the revealed optimum sulfomethylation reaction, the lignosulfonate (LG) produced from AL and EHR were termed as AL-LG and EHR-LG, respectively. The liquor of AL-LG and EHR-LG were dialyzed using dialysis membrane (molecular weight cutoff of 1000 g/mol) for 48 h (exchanging water every 12 h) to separate the lignosulfonates and un-reacted chemicals (sodium hydroxide, sodium sulfite, and formaldehyde). The dialyzed samples were then lyophilized to obtain lignosulfonate powder.

### 2.3. Measurement of Molecular Weight

The molecular weight of CM-LG, AL-LG, and EHR-LG were determined by gel permeation chromatography (Waters 1515) instrument equipped with Ultrahydragel 120 and 250 columns. The column was operated at ambient temperature and eluted with sodium nitrate (0.1 mol/L) at a flow rate of 0.5 mL/min. Sodium polystyrene sulfonate was used as calibration standards.

### 2.4. Sulfonic Group Analysis

The sulfonic group contents of CM-LG, AL-LG, and EHR-LG were analyzed by potentiometric titration. Specifically, CM-LG, AL-LG, and EHR-LG were transformed into lignosulfonic acid by passing then through anion and cation exchange resins. After ionic exchange, a certain volume (*V*_1_, mL) of lignosulfonic acid solution was titrated with 0.01 M NaOH. The pH of the solution increased with increasing consumption of NaOH (*V*_2_, mL) until the differential curve showed a distinctive mutation. The concentration (*C*_1_, g/L) of lignosulfonic acid solution was determined by an UV spectrophotometer [24]. The content of sulfonic groups in each lignosulfonate was then calculated by the equation

(2) $$S(\mathrm{mmol}/g)=\frac{0.01\times V_{2}\times 1000}{V_{1}\times C_{1}}\times 100\%$$

### 2.5. Measurement of Surface Tension

The surface tension of CM-LG, AL-LG, and EHR-LG solutions (1 g/L–10 g/L) were measured by the Wilhelmy plate method using a tension meter (DCAT21, Filderstadt, Germany). Measurements were conducted at 25 °C and pH 8. Three replicates for each sample were carried out and the average results are reported.

### 2.6. Zeta Potential Analysis

Zeta potentials of CM-LG, AL-LG, and EHR-LG solutions (0.5 g/L) were measured using a Nano-Zetasizer (ZSE, Malvern, UK). Specifically, the lignosulfonate powder was dissolved into solutions of different pH (2.5, 5.0, 7.0, 9.0, and 11.0). Next, 1 mL lignosulfonate solution was syringed into the electrophoresis cell. The zeta potential of each lignosulfonate solution was then measured after the solution in the electrophoresis cell was equilibrated for 60 s. Three replicates for each sample were performed, and the results reported are the average.

### 2.7. Effects of Lignosulfonate on Performance of Cement Paste and Concrete

The effects of lignosulfonate (CM-LG, AL-LG, and EHR-LG) on the fluidity of cement paste was determined according to the Chinese National Standard of GB 8077-200. Specifically, 300 g of the cement with 120 g of water and different dosages (0.1–0.8 wt %) of lignosulfonate were mixed for 4 min using a paste blender. The cement paste was put into a small flow cone mold on a smooth glass plate. Then the cone was lifted to collapse and spread the cement paste. When the spread of cement paste halted, the maximum diameter of the spread (*d*_1_, mm) and the diameter perpendicular (*d*_2_, mm) were measured. Fluidity of each cement paste was calculated from the average value of *d*_1_ and *d*_2_, ((*d*_1_ + *d*_2_)/2). Two replicates for each sample were carried out and the results reported are the average of the two replicates. Concrete was prepared using 4.96 kg cement, 10.66 kg silica sand, 6.84 kg small stones (5–19 mm), 10.26 kg medium stones (10–20 mm), different dosages (0.1–0.3 wt %) of lignosulfonate and water. The ingredients were mixed using a mechanical mixer, with mixing for 10 min at room temperature. The dosages of lignosulfonate (CM-LG, AL-LG, and EHR-LG) were 0.1, 0.2, and 0.3%. The water reduction of lignosulfonate in concrete was determined according to the Chinese National Standard of GB/T 50080. Entrained air in the concrete was measured according to the Chinese National Standard of GB J 80-85 using a pressure type “B” meter. The compressive strength of the concrete was tested according to the Chinese National Standard of GB 11837-89. The mixture of concrete was cast into cube plastic vials (10 × 10 × 10 cm) and compacted with an iron rod until all entrapped air was removed. The prepared samples were kept at 25 °C. After 3, 7, and 28 days of curing, three specimens per mixture were removed from the cube plastic vials and tested for compressive strength using a compression machine. Relative compressive strength was obtained by the compressive strength of concrete without lignosulfonate which was measured as a control.

## 3. Results and Discussion

### 3.1. Effect of Sulfomethylation on Lignosulfonate Production from AL and EHR

**Scheme 1:**
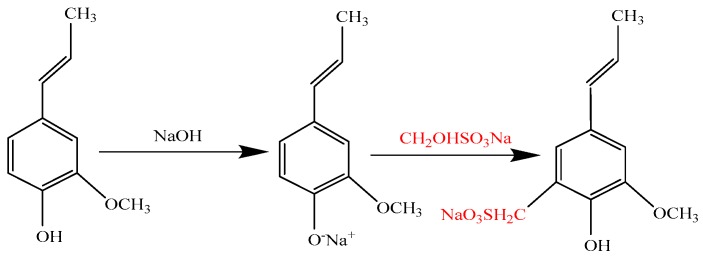
The sulfomethylation scheme of lignin.

**Figure 1:**
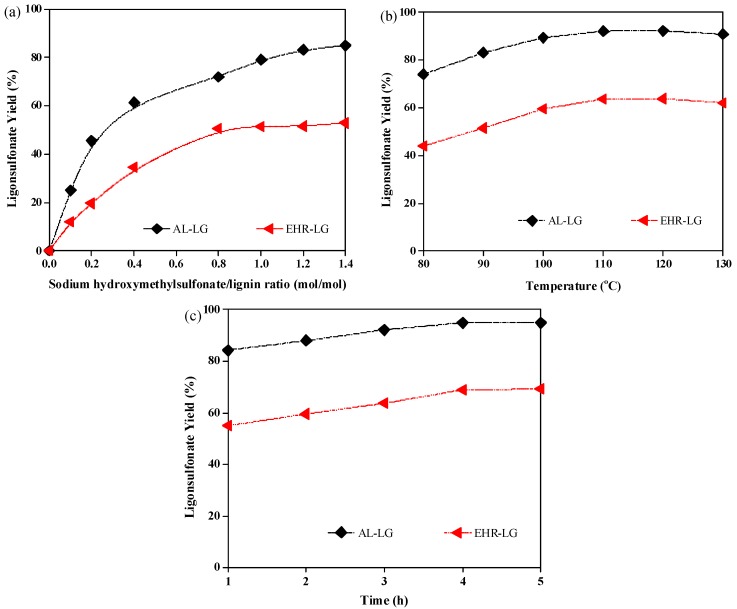
The effect of CH_2_OHSO_3_Na/lignin ratio (**a**), temperature (**b**), and reaction time (**c**) on the lignosulfonate yields of lignosulfonate (LG) from alkaline lignin (AL) and enzymatic hydrolysis residue (EHR), termed as AL-LG and EHR-LG, respectively.

For the reaction of lignin sulfomethylation, HCHO and Na_2_SO_3_ are the precursor chemicals which combine to form the reactive species, sodium hydroxymethylsulfonate [17]. The reactive species is responsible for introducing a sulfonic group onto the 5th position in the benzene ring (C5) of lignin aromatic rings, resulting in lignosulfonate. The sulfomethylation reaction scheme is show in Scheme 1. It is reported that the dosage of sodium hydroxymethylsulfonate, temperature, and reaction time are the parameters which affect the degree of sulfonation [17,18]. Hence, the impacts of different sodium hydroxymethylsulfonate/lignin ratio (0.1–1.4 mol/mol), different temperatures (80–130 °C), and different times (1–5 h) on lignosulfonate yields was investigated. Lignosulfonate yields for AL and EHR are shown in Figure 1a–c, respectively.

Firstly, it can be observed from Figure 1a that increasing the ratio of sodium hydroxymethylsulfonate/lignin lead to increases in lignosulfonate production for both AL and HER. For AL, increasing the ratio from 0.1 mol/mol to 1.2 mol/mol resulted in an increase to lignosulfonate production from 25.1% to 83.1%. However, increasing the sodium hydroxymethylsulfonate addition from 1.2 to 1.4 mol/mol did not have an obvious effect on more lignin in AL been sulfonated, with an increase in yield of only 83.1% to 83.9%. For EHR, the optimum sodium hydroxymethylsulfonate/lignin ratio was 0.8 mol/mol, resulting 50.6% yield of lignosulfonate. The bottleneck of sulfomethylation reactions upon AL and EHR may be due to a finite amount of reaction sites (free C5 position) in each respective lignin [24]. Hence, it was speculated that sodium hydroxymethylsulfonate/lignin ratios with 1.2 mol/mol and 0.8 mol/mol were the optimum conditions for AL and EHR to produce lignosulfonates.

The impact of sulfomethylation reaction temperature on lignosulfonate production can be seen in Figure 1b. It was found that when the reaction temperature was increased from 80 °C to 110 °C, the amount of lignosulfonate genesis from AL and EHR both increased. However, after 120 °C, sulfomethylation yield slightly decreased. For example, increasing temperature from 120 °C to 130 °C led to a decrease to the amount of lignosulfonate from AL and EHR (92.3% to 90.8%, and 63.9% to 62.1%, respectively). This observation may be due to the formation of the undesirable products (sodium thiosulfate), which is formed from a side reaction between HCHO and Na_2_SO_3_ at high temperatures. This observation was in agreement with the report by Pang et al. [26], which indicated that more sodium thiosulfate was formed during the sulfomethylation process of hardwood kraft lignin when sulfomethylation temperatures increased from 120 °C to 150 °C. The net effect here is less reactive species combining with lignin. Therefore, it can be inferred that higher sulfomethylation temperatures do not favor production of lignosulfonates. Finally, it was observed 110 °C was the optimum temperature for sulfomethylation of AL and EHR to produce lignosulfonates.

As shown in Figure 1c, reaction time also has significant impact on lignosulfonate yields. When the time was extended from 1 h to 3 h (for AL) and from 1 h to 4 h (for EHR), the amount of lignosulfonate production from AL and EHR increased from 84.3% to 94.9% and 54.9% to 68.9%, respectively. However, further prolonging the reaction time had no effect on lignosulfonate yield. This indicated that 3 h and 4 h reaction time at 110 °C was sufficient to produce lignosulfonates from AL and EHR using sulfomethylation. In the previous work by Wu et al. [27], they also observed extended reaction times (2 h to 5 h) had no effect on sulfomethylation of, in this case, alkalki lignin.

In all, it was found that the lignin in AL was more prone to lignosulfonate production through sulfomethylation treatment. This indicates that AL has more reaction sites (free C5 position of lignin) than EHR, possibly due to is lower molecular weight and lower condensation. Integrating all parameters, sulfomethylation reaction at 110 °C, sodium hydroxymethylsulfonate/lignin with 1.0 mol/mol and 3 h were found to be optimal conditions for lignosulfonate production from AL (94.9% yield). Differently, the optimum conditions for EHR to produce lignosulfonate were 110 °C, sodium hydroxymethylsulfonate/lignin with 0.8 mol/mol and 4 h, resulting in 68.9% lignosulfonate yield. The optimally-produced liganosulfates from the AL and EHR are henceforth termed as AL-LG and EHR-LG.

### 3.2. Molecular Weight and Sulfonic Group of Lignosulfonate

To better understand the molecular differences between the lignosulfonates obtained lignosulfonate from AL and EHR, both molecular weight and sulfonic group of AL-LG and EHR-LG were characterized. Results from these lignosulfonates were also compared to commercial lignosulfonate (CM-LG), shown in Table 1.

As seen in Table 1, the Mw and Mn of each lignosulfonate were slightly dissimilar. For the difference of molecular weight of lignosulfonate, it is important to point out that the commercial lignosulfonate was derived from wood, therefore some differences can be attributed to dissimilar starting materials. In addition, the CM-LG is produced from the sulfite pulping process, which is different from the sulfomethylation process for producing AL-LG and EHR-LG. Regarding molecular weight differences between the commercial woody sulfite lignosulfonate and the non-woody sulfomethylated lignosulfonates produced, inter-lignin condensation by way of formaldehyde is a driver of molecular weight that is unique to the sulfomethylated lignosulfonates [24,28]. However, it seems from the results that this reaction did not lead to a pronounced increase in molecular weight for either sulfomethylated sample. A possible advantage to the bamboo lignosulfonates involves PDI, where the PDI of CM-LG (4.0) was found to be higher than that of AL-LG (2.5) and EHR-LG (2.3). This indicated that the sulfomethylated bamboo lignosulfonate was more homogenous than commercial lignosulfonate, which can possibly translate to application benefits (depending on the application of course). In the work of He and Fathhi [18], they also found that the lignosulfonate produced from sulfomethylation of softwood kraft lignin exhibited good homogeneity with polydispersities of 1.2–1.8.

Concerning the sulfonic group data from Table 1, it can be seen that AL-LG possessed greater abundance of sulfonic moieties than HER-LG (1.6 vs. 1.0 mmol/g), but was quite similar to the commercial lignosulfonate (1.5 mmol/g). This indicates that introduction of sulfonic groups occurred to a greater extent for AL, which is in agreement with the results from Figure 1 and its ensuing conjecture. It is reported that more sulfonic groups in lignosulfonates result in greater anionic charge density, which are expected to translate to improved performance as water reducers for cement admixtures [17,18]. Therefore, from these results, we speculate that the lignosulfonate produced from AL will be superior in said application relative to lignosulfonate from EHR.

This section may be divided by subheadings. It should provide a concise and precise description of the experimental results, their interpretation, as well as the experimental conclusions that can be drawn.

### 3.3. Surface Tension of Lignosulfonate Solutions

**Figure 2:**
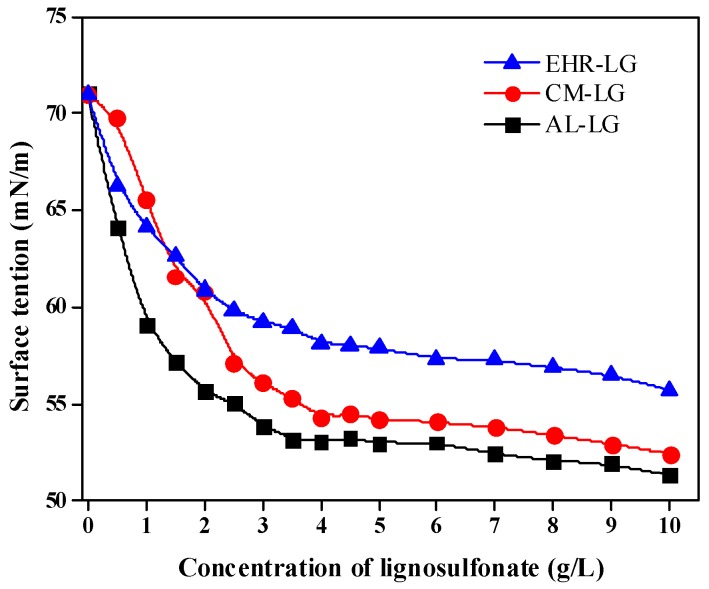
Surface tension of the lignosulfonates AL-LG, EHR-LG, and CM-LG in solutions of different concentrations.

Figure 2 shows the surface tension of AL-LG, EHR-LG, and CM-LG solutions with different concentrations (pH 8). It can be seen from the results that surface tension of lignosulfonate solutions significantly decreased with increases to concentration. Next, it can also be observed that the surface tension of the AL-LG and CM-LG were similar, which was lower than that of EHR-LG solutions when comparing at same concentration. Specifically, the surface tensions (at 5 g/L) of AL-LG, CM-LG, and EHR-LG and were 52.9, 54.2, and 57.4 mN/m, respectively. A possible explanation for these differences may be due to the quantity of sulfonic groups in AL-LG (1.6 mmol/g) was similar to CM-LG and higher than EHR-LG (1.0 mmol/g). Matsushita et al. [29] also found that the increased levels of sulfonic groups in lignosulfonate resulted in reduced surface tensions in solution.

It was also found that the surface tension of lignosulfonate solution decreased with the increasing lignosulfonate molecular weight. This can be explained by the effect that higher molecular weight of lignosulfonates have on static repulsive forces between hydrophilic groups in solution. The net effect of this is that higher molecular weight lignosulfonates form a more-dense adsorption layer at the surface. The increased density of this adsorption layer promotes higher surface activity of lignosulfonate, i.e., lower surface tension. Ouyang et al., [19] reported that the lower surface tension of lignosulfonate solutions is favorable towards cement particle dispersion into water, resulting in improved workability and fluidity of cement admixtures. Hence, it is again speculated that the lignosulfonate produced from AL by sulfomethylation will show better performance in concrete properties than that from EHR sulfomethylation.

### 3.4. Zeta Potential of Lignosulfonate Solutions

**Figure 3:**
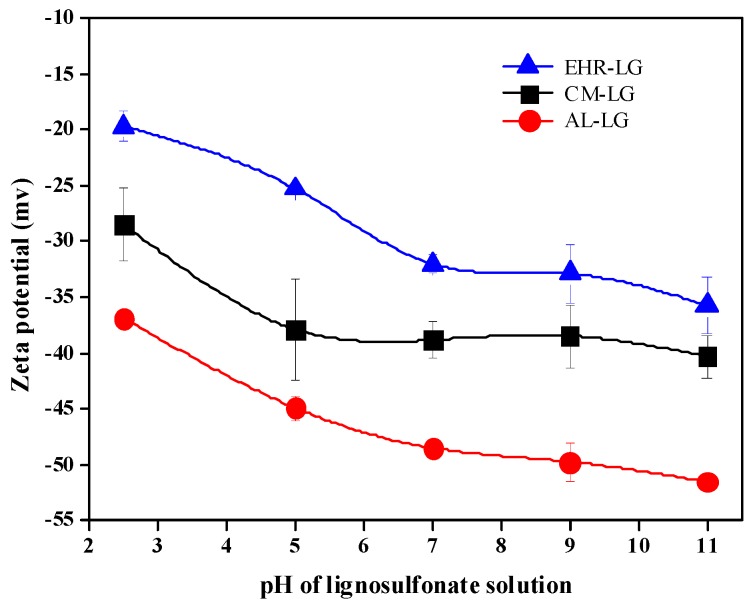
The zeta potential of lignosulfonate solutions across pH.

For lignosulfonate, the presence of sulfonic and carboxyl functional groups imbibes it with an electromotive force, which can be estimated as zeta potential. It is reported that lower zeta potentials for lignosulfonate solutions contributes to cement dispersion efficiency [19]. For this reason, the differences in zeta potentials for AL-LG, EHR-LG, and CM-LG were analyzed, with results shown in Figure 3.

It can be seen that the zeta potential of three lignosulfonate solutions decreased when increasing pH from 2.5 to 7, which might be mainly caused by ionization of sulfonic group. Moving upward on the pH scale, zeta potential of all three lignosulfonate solutions was almost unchanged when pH was raised from 7 to 9. This indicated that the zeta potential of lignosulfonate is affected by the pH of its solution [22]. For cement paste, the pH is mildly alkaline (pH 8–9) and the zeta potential of cement particle surfaces is positively charged (~10 mV). Furthermore, miniscule electrostatic repulsion occurs between only wetted cement particles, leading to unstable suspensions of wet cement [30]. When lignosulfonate is added to cement paste, it can be adsorbed on the surface of cement particles, resulting the changes of the zeta potential of the particles. This change enables electrostatic repulsion between lignosulfonate-coated cement particles, thus stabilizing the cement dispersion. From Figure 3, it was found that the zeta potential of AL-LG solution was lower than that of EHR-LG and CM-LG. Specifically the zeta potentials of AL-LG, EHR-LG, and CM-LG solutions at pH 8 (the pH of cement paste) were −49.4, −29.8, and −39.6 mV, respectively. The difference in the zeta potential of each different lignosulfonate is most likely driven by different amounts of sulfonic groups, molecular weight and the geometry of lignosulfonates in solution [27]. As both AL-LG and EHR-LG possessed the negative zeta potentials similar to commercial lignosulfonate (CM-LG), we expect them both to have functionality as cement dispersants/water reducers. Based on these results, it is likely that AL-LG will be the highest performing water reducer of the two biorefinery lignosulfonates prepared.

### 3.5. Evaluation of Lignosulfonates as Cement Paste Dispersants

**Figure 4:**
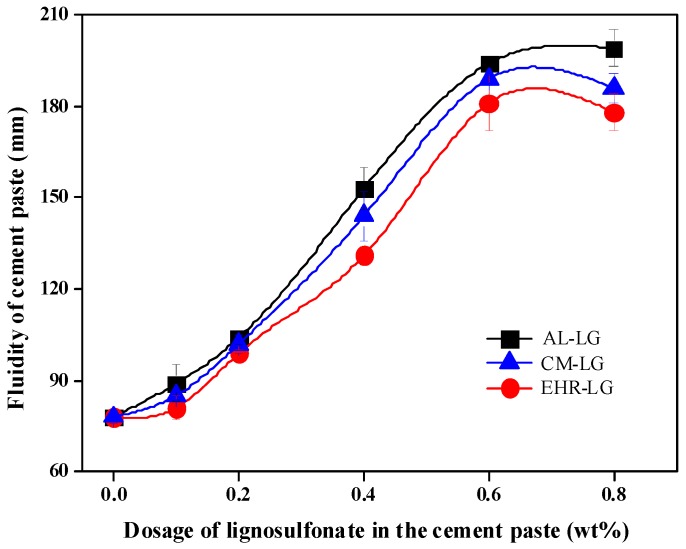
The effects of AL-LG, EHR-LG, and CM-LG on the fluidity of cement paste.

Evaluation results from the tested lignosulfonates (two biorefinery lignosulfonates vs. commercial material) are shown in Figure 4. From the results shown it can be seen that the fluidity of cement paste increased with increased dosages of lignosulfonate in the ranges of 0.1 wt % to 0.6 wt %. However, it was also observed that fluidity of cement paste slightly decreased when the addition of CM-LG and EHR-LG increased from 0.6 wt % to 0.8 wt %. It was also found that the fluidity of cement paste blended with AL-LG was always slightly better than that with EHR-LG or CM-LG. One reason for this observation is due to the lower zeta potential measured for AL-LG. For the dispersant with lower zeta potential, it can create a stronger electrostatic repulsive force between cement particles, which is respond for the better fluidity of cement paste [23,31]. Steric hindrance is also a factor which contributes to improvement in cement paste fluidity, which is positively affected by the molecular weight of the applied dispersant [32]. As shown in Table 1, the molecular weight of AL-LG was 15,470 g/mol, higher than that of EHR-LG (10,230 g/mol) and CM-LG (12,180 g/mol). Hence, higher molecular weight of AL-LG may be another reason for the better fluidity of cement paste blended with AL-LG. In the work of Ouyang et al. [19], Konduri and Fatehi [17], they also found that fluidities of cement pastes were increased when prepared with lignosulfonates of greater molecular weight and lower zeta potential. Meanwhile, Figure 4 showed that the addition of CM-LG in cement paste can also result considerable positively effect on its fluidity, compared to the cement paste with AL-LG and EHR-LG. These results suggest that both the AL-LG and EHR-LG can be used as the dispersant to improve the fluidity of cement paste.

### 3.6. Effect of Lignosulfonate on the Concrete Properties

The effectiveness of lignosulfonate additives in concrete admixtures is not only determined gauged by improvements to cement paste fluidity, but also determined by its effects upon water reduction, air entrainment, and the resultant compressive strength of finished concrete [20]. Therefore, the effects of addition of AL-LG and EHR-LG on these properties of concrete were investigated, with the results contained in Table 2.

From Table 2, it can be seen that all three lignosulfonates benefitted water reducing ratio, of which AL-LG showed best performance at the dosage of 0.1–0.2 wt %. EHR-LG demonstrated the best water reducing performance at 0.3 wt % dosage, with a water reduction ratio of 14.1% These results indicate that the produced lignosulfonates from biorefinery lignins by sulfomethylation showed performance with higher water reduction as water reducers for concrete compared to the commercial lignosulfonate.

Table 2 showed results of the finished concrete products with addition of three lignosulfonates at different dosages. It can be seen that 0.1 wt % and 0.2 wt % of each tested lignosulfonate improves the 28 day compressive strength of finished concrete at each time interval measured. While, the 28 day compressive strengths of the concretes declined slightly with the addition of 0.3 wt % lignosulfonates. For example, the compressive strengths of concretes were decreased from 41.6 Mpa to 36.4 Mpa and from 42.6 Mpa to 38.2 Mpa when addition of AL-LG and EHR-LG increased from 0.2 wt % to 0.3 wt %, respectively. This indicated that both lignosulfonates showed best performance in improving the compressive strength of concrete with the dosage of 0.2 wt %. This observation may be due to greater air entrainment when more lignosulfonate was added for improving the fluidity of cement [33]. As lignosulfonate in the concrete can not only absorb on the solid–liquid interface, but also on the solid–gas interface, resulting the forming of some air bubbles in the finished concrete as it ages. Trapped bubbles can generate pores in the concrete during the solidification process, thereby decreasing its compressive strength [19,20]. Exactly as the results of air content in Table 2, with the addition of AL-LG and EHR-LG increased from 0.2 wt % to 0.3 wt %, the air content in the concrete was found to increase from 4.0% to 6.2% and from 3.0% to 5.3%, respectively. In the work of Ouyang et al. [19], they also found that increasing the air content resulted the more pores in the concrete, which resulted in decreases to compressive strength.

As discussed before, AL-LG showed better performance in improving the fluidity of cement paste and reducing the consumption water of concrete than EHR-LG. However, EHR-LG had a more positive effect on the compressive strength of concrete than that of AL-LG (albeit only when the dosage was between 0.2 wt % and 0.3 wt %). This may also due to more air content was trapped into the concrete with the addition of AL-LG. Hence, it can be speculated that the lignosulfonate with superior properties of water reduction may do not favor to enhance the compressive strength of concrete, due to more air bubbles may be trapped with the better fluidity of cement. Overall, the results from the concrete analysis suggested that the lignosulfonate from biorefinery lignins by sulfomethylation with the addition of 0.1–0.2 wt % have met industrial standards as a water reducer for improving the properties of concrete, which is slightly better than the commercial lignosulfonate.

## 4. Conclusions

A systematic investigation revealed that a lignosulfonate yield with 94.9% and 68.9% can be realized from AL (AL-LG) and EHR (EHR-LG) by sulfomethylation under optimum conditions, respectively. The sulfonic groups in AL-LG and EHR-LG were 1.6 mmol/g and 1.0 mmol/g, respectively. Compared to EHR-LG and commercial lignosulfonate (CM-LG), AL-LG showed the best performance in improving the fluidity of cement paste due to its lowest surface tension and zeta potential. With addition of these lignosulfonates in concrete, AL-LG showed best performance regarding achieve water reducing ratio. EHR-LG was found to uniquely increase the compressive strength (28 days) of concrete.

## Figures and Tables

**Table 1:** Weight-average molecular weight (Mw), number-average molecular weight (Mn) and sulfonic group of AL-LG, EHR-LG, and CM-LG ^1^.

Lignosulfonate	Mw (g/mol)	Mn (g/mol)	PDI ^2^	Sulfonic Group (mmol/g)
AL-LG	15,470	6120	2.5	1.6
EHR-LG	10,230	4440	2.3	1.0
CM-LG	12,180	3060	4.0	1.5

^1^ CM-LG, lignosulfonate (LG) from commercial lignosulfonate (CM); ^2^ polydispersity, Mw/Mn.

**Table 2:** The effects of addition of AL-LG, EHR-LG, and CM-LG on these properties of concrete

Lignosulfonate	Dosage (wt %) ^1^	Water Reduction (%)	Air Content (%)	Compressive Strength (Mpa)		
				7 Days	14 Days	28 Days
None ^2^	/	/	/	26.7	33.4	38.4
AL-LG	0.1	5.8	2.1	28.5	36.3	40.9
	0.2	10.6	4.0	29.5	35.2	41.6
	0.3	13.6	6.2	24.5	34.2	36.4
EHR-LG	0.1	3.8	2.0	28.7	36.4	39.7
	0.2	6.0	3.0	26.4	36.6	42.6
	0.3	14.1	5.3	26.6	33.6	38.2
CM-LG	0.1	1.6	2.3	27.9	34.4	38.0
	0.2	3.9	3.0	28.0	33.9	40.9
	0.3	6.8	5.0	24.2	35.3	37.8

^1^ Based on the amount of cement in the concrete; ^2^ Concrete with no lignosulfonate.
